# Optimizing tissue lysis and DNA extraction protocols to enhance bacterial diversity profiling in the *Drosophila melanogaster* gut microbiome

**DOI:** 10.1038/s41598-026-63122-w

**Published:** 2026-07-30

**Authors:** Carlos L. Quiñones-Sanchez, Jan L. Bilbao-Del Valle, Miguel A. Urdaneta-Colon, Tasha M. Santiago-Rodriguez, Imilce A. Rodriguez-Fernandez

**Affiliations:** 1 Department of Biology, College of Natural Sciences, University of Puerto Rico, Rio Piedras, San Juan, 00925 Puerto Rico; 2 The Alkek Center for Metagenomics and Microbiome Research, Department of Molecular Virology and Microbiology, Baylor College of Medicine, Houston, TX 77030 USA; 3 Department of Molecular Virology and Microbiology, Baylor College of Medicine, Houston, TX 77030 USA

**Keywords:** DNA extraction, 16S rRNA, Gut microbiome, *Drosophila melanogaster*, Microbial diversity, Biological techniques, Biotechnology, Computational biology and bioinformatics, Microbiology

## Abstract

The gut microbiota is a dynamic community that influences host metabolism, immunity, and overall health. Accurate characterization of this community requires robust and reproducible DNA extraction methods; however, technical biases introduced during tissue lysis and DNA isolation remain major challenges in microbiome research, particularly in animal model systems. In this study, we compared two lysis methods (manual pestle homogenization and bead-beating) and two commercial DNA extraction kits (Qiagen and Zymo) to evaluate their impact on microbiota profiling in a microbial community standard (MCS) and *Drosophila melanogaster* gut samples, a tractable model for host–microbe interactions. Full-length 16S rRNA sequencing was performed using Oxford Nanopore Technologies (ONT), followed by taxonomic classification with EPI2ME and downstream diversity analyses using standard pipelines implemented through a custom script. Our data revealed that extraction and lysis methods influence microbial composition, with some protocols resulting in additional species-level assignments in MCS samples, likely reflecting limitations of sequencing technology and taxonomic misidentifications. Pestle homogenization, combined with the Qiagen kit, recovered the highest number of detected taxa and showed a more balanced representation of both Gram-positive and Gram-negative bacteria across the *Drosophila* gut samples. These findings highlight that extraction methodology influences microbial diversity estimates in *Drosophila* gut samples, although sequencing approach and taxonomic classification accuracy may also contribute to the observed differences. Our results emphasize that the choice of DNA extraction protocol should be guided by the biological question being addressed and highlight the need for standardized protocols to ensure reproducibility across microbiome studies, particularly those using model systems.

**Supplementary Information:**

The online version contains supplementary material available at https://doi.org/10.1038/s41598-026-63122-w.

## Introduction

The microbiome, often referred to as an “unseen organ”, encompasses the diverse communities of bacteria, fungi, viruses, archaea, and eukaryotes that inhabit host organisms and interact with one another. These microorganisms play essential roles in maintaining physiological balance, regulating immunity, synthesizing metabolites and amino acids, and providing protection against pathogens^1–5^. Factors such as age, diet, infection, and antibiotic exposure can disrupt this balance, leading to microbiome dysbiosis and associated health disorders^3,4^.

Advances in sequencing technologies have transformed microbiome research. Whole-genome sequencing (WGS) enables species-level resolution and functional profiling, while 16S rRNA gene sequencing (16S) remains a cost-effective method for taxonomic characterization^5–7^. Traditional short-read approaches typically target partial variable regions (e.g., V3-V4), which generally provide genus-level resolution while limiting accurate species-level classification^8^. Full-length 16S sequencing (~ 1,500 bp) overcomes this limitation by typically improving species-level resolution and accuracy^6,8,9^.

Long-read sequencing technologies now permit near full-length 16S amplicon sequencing. Oxford Nanopore Technologies (ONT) and PacBio platforms generate reads spanning the entire 16S gene, improving species-level resolution. In this study, we employed ONT due to its accessible, cost-effective workflows and suitability for laboratories with limited computational infrastructure^10–15^.

ONT also offers integrated, user-friendly solutions such as the EPI2ME cloud platform for demultiplexing, taxonomic assignment, and diversity analysis, allowing users with limited computational experience to process data without high-performance computing resources and programming expertise^16^. This accessibility makes ONT particularly suitable for microbiome studies in small or resource-limited laboratories.

Accurate microbiome profiling depends not only on sequencing technology but also on effective sample lysis and DNA extraction. The efficiency of cell disruption during these steps is critical as microbial cells in complex materials, such as host tissues, can be difficult to lyse. Suboptimal extraction can bias species detection, particularly for Gram-positive bacteria with thick peptidoglycan walls^17–21^. Methodological differences at this step can therefore influence downstream diversity metrics and taxonomic profiles.

Methodological biases introduced during DNA extraction have been widely documented in human and environmental microbiome studies (reviewed in^22–25^). However, comparatively fewer studies have systematically evaluated how differences in tissue lysis and extraction chemistry influence microbiome profiling outcomes in insect systems. In the fruit fly *Drosophila melanogaster*, most microbiome studies employ a single extraction workflow and therefore, do not directly test how alternative lysis or extraction strategies may affect community readouts (reviewed in^26,27^).

Although most microbiome studies focus on humans or murine models, *D. melanogaster* has emerged as a powerful system for dissecting host-microbiome interactions^27–29^. The fly gut microbiota is relatively low in complexity, typically comprising ~ 5–20 species in laboratory-reared strains and, dominated by genera such as *Acetobacter* (Gram-negative), and *Lactobacillus* (Gram-positive) (^30^; reviewed in ^26,28,31^). The ability to generate germ-free and gnotobiotic flies enables precise control over microbial composition while simultaneously leveraging powerful genetic tools to manipulate host pathways (reviewed in ^31,32^). Together with its short life cycle, these features make *Drosophila* a powerful model for testing how methodological workflows and interventions influence the gut microbiome composition and abundance. Mechanistic studies have demonstrated that specific bacterial taxa modulate host developmental timing (^33,34^; reviewed in ^32^), nutrient metabolism ^34–39^, immune signaling pathways (^40,41^; reviewed in ^28^), lifespan ^42^, aging ^43,44^, and behaviors such as mating preference ^45^ and locomotion ^46^.

In this study, we compared four protocols combining two tissue homogenization and two commercial DNA extraction kits to determine which yields the most comprehensive and reproducible bacterial profiles in *Drosophila* gut samples and a commercially available microbial community standard (MCS). By integrating alpha and beta diversity analyses, taxonomic composition, and Gram-stain classification, we evaluated how extraction protocols influence observed taxonomic profiles relative to the expected composition of a defined MCS. In addition, we assessed the consistency and reproducibility of taxa detection in *Drosophila* gut samples.

This optimized workflow provides a practical framework for reliable, Nanopore-based microbiome analyses in low-resource laboratory environments without access to high-performance computing facilities and underscores the importance of protocol standardization to ensure consistency and comparability across studies.

## Results

### Taxonomic classification of the mock community revealed species-level overclassifications

We compared the performance of two commercially available DNA extraction kits and two tissue homogenization methods in recovering representative bacterial DNA from *D. melanogaster* gut samples (Table 1). The four protocols—BPQK, BPZK, ZBQK, and ZBZK—represent combinations of Blue Pestle (BP) or Zymo Bead Beating (ZB) homogenization with the Qiagen DNeasy Blood & Tissue Kit (QK) or the ZymoBIOMICS™ DNA Miniprep Kit (ZK), respectively. To evaluate the extraction performance, an MCS consisting of five Gram-positive, and three Gram-negative bacterial species present at equal abundance (12% each), along with two yeast species (2% each), was included as a control. The MCS underwent the same tissue lysis and genomic DNA (gDNA) extraction procedures as the *Drosophila* samples.

**Table 1:** Overview of DNA extraction protocols used in this study.

Sample ID	Sample Type	Replicates	Homogenization Method	Extraction Method	Sequencing Run
BPQK	Microbial Community Standard (MCS)	4	Blue Pestle	Qiagen DNeasy Blood & Tissue Kit	Run 1, Run 2
	*Drosophila* guts	3 (10 guts per replicate)			Run 1, Run 2
ZBQK	Microbial Community Standard	4	Bead Beating	ZymoBIOMICS™ DNA Miniprep Kit	Run 1
	*Drosophila* guts	3 (10 guts per replicate)			
BPZK	Microbial Community Standard	4	Blue Pestle	Qiagen DNeasy Blood & Tissue Kit	Run 2
	*Drosophila* guts	3 (10 guts per replicate)			
ZBZK	Microbial Community Standard	4	Bead Beating	ZymoBIOMICS™ DNA Miniprep Kit	Run 2
	*Drosophila* guts	3 (10 guts per replicate)			

**Fig. 1:**
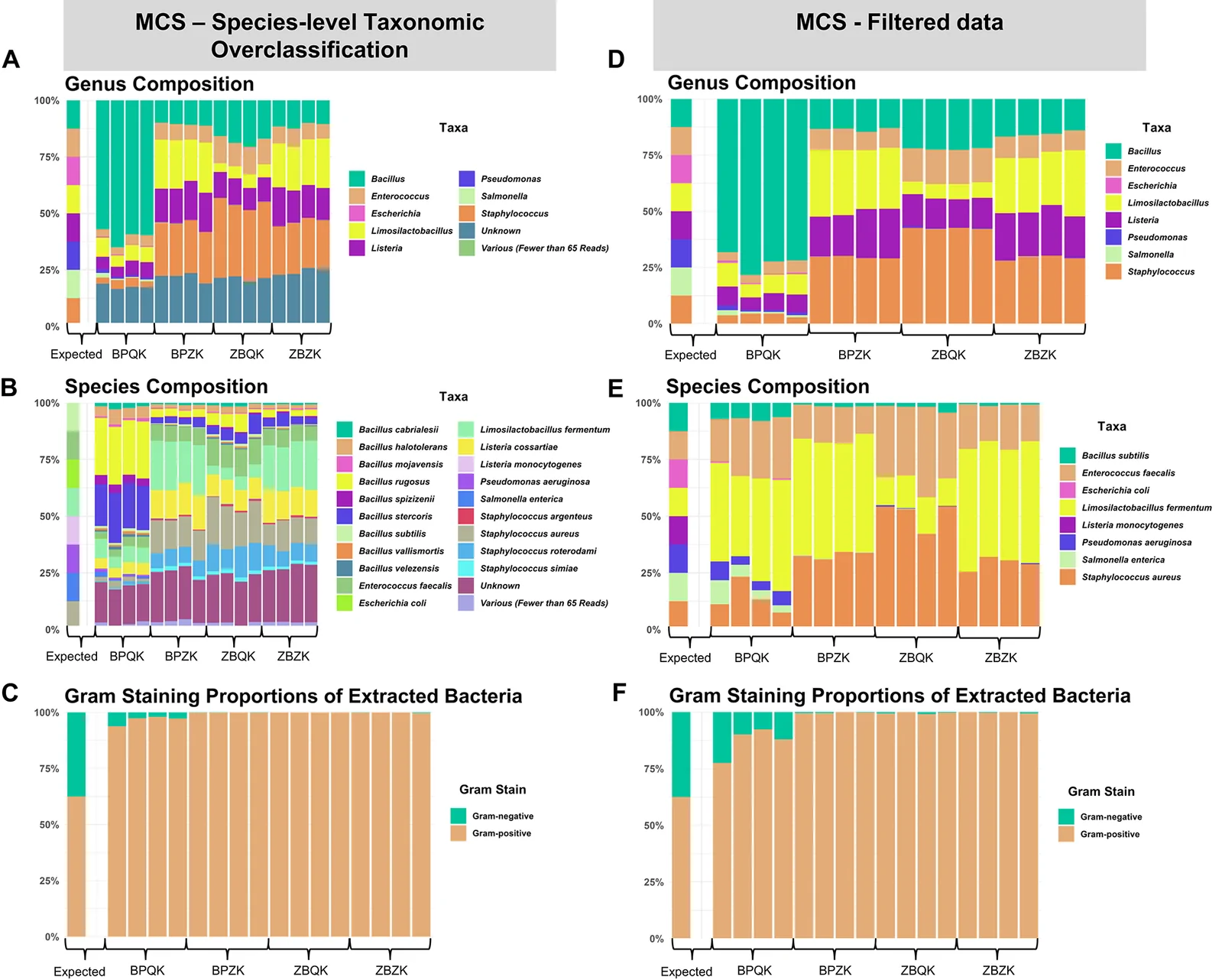
Taxonomic composition and Gram-stain distribution of the microbial community standard (MCS) across four DNA extraction protocols. **(A–C)** *Unfiltered species-level assignments data.* Full-length 16S rRNA sequencing of the MCS after DNA extraction using four protocol combinations (BPQK, BPZK, ZBQK, and ZBZK), representing Blue Pestle (BP) or Zymo Bead Beating (ZB) homogenization paired with the Qiagen DNeasy Blood & Tissue Kit (QK) or Zymo DNA Miniprep Kit (ZK). **(A)** Genus-level composition showing the expected and observed distributions. **(B)** Species-level composition revealing apparent overclassification of species-level assignments. **(C)** Gram-stain proportions inferred from species assignments, showing an apparent dominance of Gram-positive taxa. (**D–F)** *Filtered data.* Taxonomic composition after bioinformatic filtering to remove misidentified taxa and retain only true positives. **(D)** Genus-level composition of the filtered dataset. **(E)** Species-level composition representing the true mock community members. **(F)** Gram-stain proportions of the filtered dataset, showing that only the BPQK protocol recovered a noticeable fraction of DNA from Gram-negative bacteria. *Abbreviations:* BPQK = Blue Pestle + Qiagen Kit; BPZK = Blue Pestle + Zymo Kit; ZBQK = Bead Beating + Qiagen Kit; ZBZK = Bead Beating + Zymo Kit.

Full-length 16S sequencing of the MCS processed with all four extraction protocols identified the expected bacterial genera across all protocols, except for *Escherichia* that was only detected in BPQK samples (Fig. 1A). Notably, a proportion of the reads were classified as “unknown” across all extraction protocols. These reads likely represent sequences that could not be confidently assigned to a particular taxon by the EPI2ME reference database after quality filtering.

However, species-level classification resulted in overclassification of taxa (herein referred to as “species-level overclassification”), with several community members misidentified or overrepresented (Fig. 1B). Because this pattern was observed across all extraction protocols, it is likely a combination of the known limitations of ONT sequencing and taxonomic classification pipelines combined with differences in extraction efficiency. This overclassification affected the inferred Gram-stain distribution, leading to an apparent dominance of Gram-positive taxa across all conditions (Fig. 1C).

To assess whether unexpected taxa resulted from laboratory or reagent contamination, the non-template control (NTC) from the initial PCR, in which the same nuclease-free water used to dilute samples replaced template DNA, was sequenced alongside all samples. The NTC yielded only two low-abundance bacterial taxa, namely *Cutibacterium acnes* and *Roseateles saccharophilus*, neither of which was detected in the MCS or *Drosophila* gut samples. However, because reagent-only extraction blanks were not included, low-level kit-derived contaminants cannot be completely excluded.

After bioinformatically removing misidentified taxa and retaining only true positives (herein referred to as “filtered MCS”), we evaluated each extraction protocol by comparing the observed taxa with the expected composition of the MCS. Importantly, this filtering step was applied exclusively to the MCS dataset for benchmarking purposes and was not applied to interpret biological samples (i.e., *Drosophila* gut samples). At the genus level, removing unexpected taxa resulted in only minor changes in the genus-level relative abundance profiles, which remained broadly similar to those observed in the unfiltered MCS dataset, (Fig. 1D) demonstrating that genus-level classifications are more robust to sequencing-related artifacts than species-level assignments. However, protocol-specific differences in genus-level relative abundance profiles, particularly for BPQK, remained evident after filtering.

For instance, in all BPQK samples, the genus *Bacillus* represented the largest proportion of reads (~ 70%), whereas other genera were detected at lower relative abundances. In contrast, *Bacillus* was less abundant in the remaining protocols, which showed higher proportions of *Staphylococcus, Listeria,* and *Limosilactobacillus*.

Species-level filtering improved recovery of expected taxa for benchmarking purposes; however, because it constrains the dataset to known taxa, it does not represent an unbiased reconstruction of the mock community composition (Fig. 1E). *Limosilactobacillus fermentum* was highly abundant across all samples and *Staphylococcus aureus* prevalent in BPZK, ZBQK, and ZBZK samples. Filtering also altered the inferred Gram-stain proportions of detected taxa (Fig. 1F); among all protocols, only BPQK recovered a noticeable fraction of DNA from Gram-negative bacteria, although this observation may also reflect technical or sequencing-related biases.

To further evaluate the taxonomic accuracy of each extraction protocol, true positives (TP), false positives (FP), and false negatives (FN) were summarized at the genus level (Table 2), and visualized using Venn diagrams at the species level (**Supplementary Fig. 1; Supplementary Data 2**). In BPQK samples, all eight expected bacterial genera were correctly identified, and was the only protocol that detected *Escherichia;* though four false positives (*Citrobacter*, *Shigella*, *Mammaliicoccus* and several unknown taxa) were also detected (Table 2). Both BPZK and ZBQK protocols recovered most expected genera, with *Escherichia* being the only false negative (Table 2). These protocols also showed the presence of *Mammaliicoccus* and *Macrococcus* as false positives. In contrast, the ZBZK protocol failed to detect both *Salmonella* and *Escherichia*, but no false positives were observed in these samples, although some unknowns were present (Table 2).

**Table 2:** Detection of expected and non-target genera across DNA extraction methods. Checkmarks (√) indicate detection of a genus in the sequencing results, whereas crosses (x) indicate that the genus was not detected. Expected genera correspond to taxa included in the mock microbial community. Summary rows report the number of true positives (expected taxa correctly detected), false negatives (expected taxa not detected), and false positives (including taxa assigned as “Unknown” and any classified genus not present in the mock community) for each extraction method. Precision, recall, and F1-score were calculated considering all expected and unexpected genera, including taxa assigned as "Unknown.

Genus	Expected	BPQK	BPZK	ZBQK	ZBZK
*Bacillus*	√	√	√	√	√
*Listeria*	√	√	√	√	√
*Staphylococcus*	√	√	√	√	√
*Enterococcus*	√	√	√	√	√
*Limosilactobacillus*	√	√	√	√	√
*Salmonella*	√	√	√	√	x
*Escherichia*	√	√	x	x	x
*Pseudomonas*	√	√	√	√	√
*Citrobacter*	x	√	x	x	x
*Shigella*	x	√	x	x	x
*Mammaliicoccus*	x	√	√	√	x
*Macrococcus*	x	x	√	√	x
Unknown	x	√	√	√	√
True positives (TP)	–	8	7	7	6
False negatives (FN)	–	0	1	1	2
False positives (FP)	–	4	3	3	1
Precision = TP / (TP + FP)	n/a	0.67	0.70	0.70	0.86
Recall = TP / (TP + FN)	n/a	1.00	0.88	0.88	0.75
F1-score = 2 × (Precision × Recall) / (Precision + Recall)	n/a	0.80	0.78	0.78	0.80

Using the MCS as a ground-truth reference, we quantified classification performance for each protocol (Table 2). Calculations included taxa assigned as “Unknown”. Precision (TP / (TP + FP)) ranged from 0.67 to 0.86, with ZBZK showing the highest precision due to fewer false positives, while BPQK showed lower precision due to increased spurious taxa detection. In contrast, recall (TP / (TP + FN)) ranged from 0.75 to 1.00, with BPQK achieving complete recovery of expected genera and ZBZK showing reduced sensitivity. Moreover, the F1-score (2 ✕ (precision ✕ recall) / (precision + recall)) showed minimal variation across protocols (0.78–0.80), indicating broadly comparable overall classification performance despite differences in precision and recall (Table 2). These results show a trade-off between recovery of expected taxa and the presence of false positives across protocols. Importantly, these error metrics reflect combined effects of sequencing, basecalling, and taxonomic classification, and therefore serve as a benchmark of pipeline performance under the current sequencing and classification framework rather than a direct measure of extraction efficiency.

At the species level, all four protocols detected over 30 false positives in their samples (**Supplementary Fig. 1**). These findings are consistent with known limitations of species-level classification in Nanopore-based full-length 16S sequencing and should be interpreted with caution. The BPQK method detected 7 of the 8 expected species, representing the highest number of detected expected taxa (true positives) among all extraction protocols (**Supplementary Fig. 1A**), whereas the remaining protocols detected only 5–6 of the expected species (**Supplementary Fig. 1B-1D**). However, differences at the species level should not be interpreted as definitive measures of extraction accuracy due to limitations in taxonomic resolution.

To evaluate the relationships between false-positives and true-positive taxa, we calculated the p-distances between the reference sequences used by EPI2ME for taxonomic classification. These relationships were visualized using a Neighbor-Joining Phylogenetic Tree constructed in MEGA12 (**Supplementary Fig. 2**). Most false positives clustered within the same genera as their corresponding true positives, with pairwise sequence identities exceeding 99% in most cases, supporting the interpretation that these misclassifications at the species-level arise from limited taxonomic resolution rather than true biological differences.

While these differences suggest variability across protocols, they should be interpreted cautiously, as compositional shifts may also reflect technical biases unrelated to extraction efficiency. Notably, species-level filtering does not recover the true composition of the mock community but instead facilitates identification of expected taxa for benchmarking purposes.

### Species-level overclassification in the mock community data led to biases in alpha and beta diversity.

**Fig. 2:**
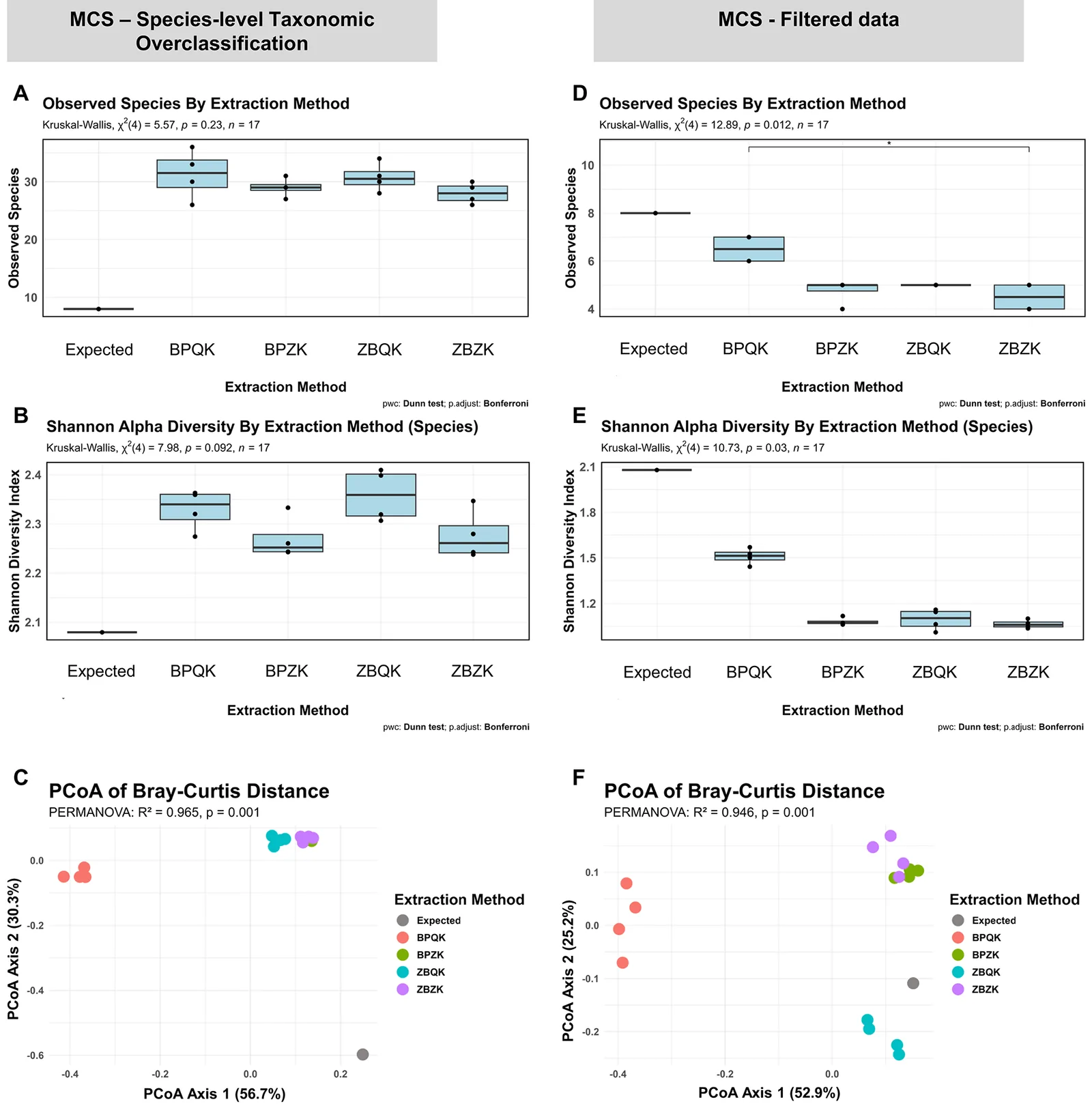
Comparison of alpha and beta diversity of the Microbial Community Standard (MCS) across four DNA extraction protocols before and after bioinformatic filtering. Panels **A–C** correspond to unfiltered data prior to removing misidentified taxa, and panels **D–F** correspond to the filtered dataset. (**A**) Observed species richness across protocols compared with the expected mock composition. (**B**) Shannon diversity index values calculated at the species level. (**C**) Principal Coordinates Analysis (PCoA) of Bray–Curtis dissimilarities displaying sample distribution by extraction method. (**D**) Observed species richness across protocols after filtering. (**E**) Shannon diversity index values of the filtered dataset calculated at the species level. (**F**) PCoA of Bray–Curtis dissimilarities displaying community distribution among extraction methods after filtering. The percentage of variance explained by each PCoA axis is indicated in the figure.

Next, we sought to demonstrate how species-level overclassification can influence diversity analyses using the MCS as a controlled example. Elevated richness in unfiltered datasets primarily reflects species-level overclassification rather than true biological diversity. To determine how species-level overclassification affected diversity estimates, we compared the observed species richness, Shannon diversity index, and Bray–Curtis dissimilarities between the MCS dataset with species-level overclassification (unfiltered) and the filtered MCS datasets relative to the expected community (Fig. 2). Before filtering, all extraction protocols showed a trend toward higher alpha diversity values than expected, as reflected by both observed species richness (Fig. 2A) and Shannon diversity index (Fig. 2B). However, differences in alpha diversity among extraction protocols were not statistically significant (Kruskal–Wallis, p > 0.05). In contrast, Bray–Curtis dissimilarities, using Bray–Curtis dissimilarity ~ group (i.e., DNA extraction and tissue homogenization methods combined) as the model, revealed significant differences among protocols and the expected community, with BPQK samples appearing to form a distinct cluster separate from the other methods and from the expected composition (R2 = 0.965, p = 0.001) (Fig. 2C). Pairwise PERMANOVA further revealed significant differences between BPQK and BPZK (R2 = 0.96, FDR-adjusted *p* = 0.028), ZBQK (R2 = 0.93, FDR-adjusted p = 0.028), and ZBZK (R2 = 0.95, FDR-adjusted *p* = 0.029). Additional significant differences were observed between BPZK and ZBQK (R2 = 0.81, FDR-adjusted p = 0.030) and between ZBQK and ZBZK (R2 = 0.76, FDR-adjusted *p* = 0.029).

Next, to illustrate how species-level overclassification can influence diversity analyses, we examined the MCS dataset after filtering taxa classified at the species level as a controlled benchmarking exercise. We noted a decrease in observed species richness and Shannon diversity across all protocols (Fig. 2D–E). Alpha diversity differed among extraction methods (Observed species: Kruskal–Wallis χ2 = 12.89, *p* = 0.012; Shannon index: χ2 = 10.73, *p* = 0.03), with BPQK showing the highest values.

Beta diversity based on Bray–Curtis dissimilarities showed apparent clustering by extraction method; BPQK appeared to form a distinct cluster, whereas BPZK, ZBZK and ZBQK clustered closest to the expected composition (Fig. 2F). PERMANOVA confirmed significant compositional differences among groups (R2 = 0.946, *p* = 0.001). Similarly, pairwise PERMANOVA showed significant differences between BPQK and BPZK (R2 = 0.66, FDR-adjusted *p* = 0.028), ZBQK (R2 = 0.87, FDR-adjusted *p* = 0.027) and ZBZK (R2 = 0.74, FDR-adjusted *p* = 0.028). Additional significant differences were noted between ZBQK and ZBZK (R2 = 0.91, FDR-adjusted *p* = 0.030).

To determine whether these differences were attributable to the tissue homogenization or DNA extraction method, we performed an additional factorial PERMANOVA using the model Bray–Curtis dissimilarity ~ tissue homogenization method + DNA extraction method. Results demonstrated that both DNA extraction and tissue homogenization methods contributed significantly to differences in mock community composition. However, DNA extraction method explained a greater proportion of the variance than tissue homogenization (R2 = 0.46 vs. 0.23; *p* < 0.001 for both factors). Similar results were obtained in the unfiltered datasets (R2 = 0.44 vs. 0.24; *p* < 0.001 for both factors).

Variability was evaluated using a test for homogeneity of multivariate dispersion based on Bray–Curtis distances; distances to centroid did not differ significantly between methods (ANOVA: F = 0.202, *p* = 0.8929; **Supplementary Fig. 3A**), indicating comparable dispersion across groups. Because Bray–Curtis dissimilarity reflects relative abundance structure rather than presence/absence recovery, proximity to the expected equal-abundance profile does not necessarily indicate superior recovery of expected taxa. Importantly, because filtered datasets are constrained to expected taxa, they are not independent representations of community composition and are used here solely for benchmarking purposes. Accordingly, because all samples originate from the same MCS, these differences do not represent true biological variation but instead reflect a combination of methodological and technical effects. In particular, compositional differences may arise from extraction-related variation as well as sequencing- and classification-related biases. Additionally, the relatively low rarefaction depth used in this study may influence diversity estimates and PERMANOVA sensitivity and should be considered when interpreting these results.

To ensure that these results were not driven by within-group dispersion, a test for homogeneity of multivariate dispersion was conducted. Distances to group centroids did not differ significantly among extraction methods for the MCS dataset (**Supplementary Fig. 3A**), showing comparable levels of variance across groups. Although BPQK recovered the highest number of expected taxa after filtering, beta diversity analysis indicated compositional deviation from the expected mock community.

To mitigate potential batch effects, identical DNA samples were included across sequencing runs; however, residual run-to-run variability (e.g., flow cell performance, library preparation, and basecalling differences) cannot be fully excluded and should be considered when interpreting compositional differences.

### *Drosophila* gut samples processed with the BPQK protocol showed broader taxonomic recovery

Next, we evaluated the performance of the four extraction protocols in recovering bacterial gDNA from *D. melanogaster* gut samples, used here as a model animal tissue commonly employed to study the effects of experimental interventions on gut microbiota composition. These gut bacterial samples were processed in parallel with the MCS samples described above.

**Fig. 3:**
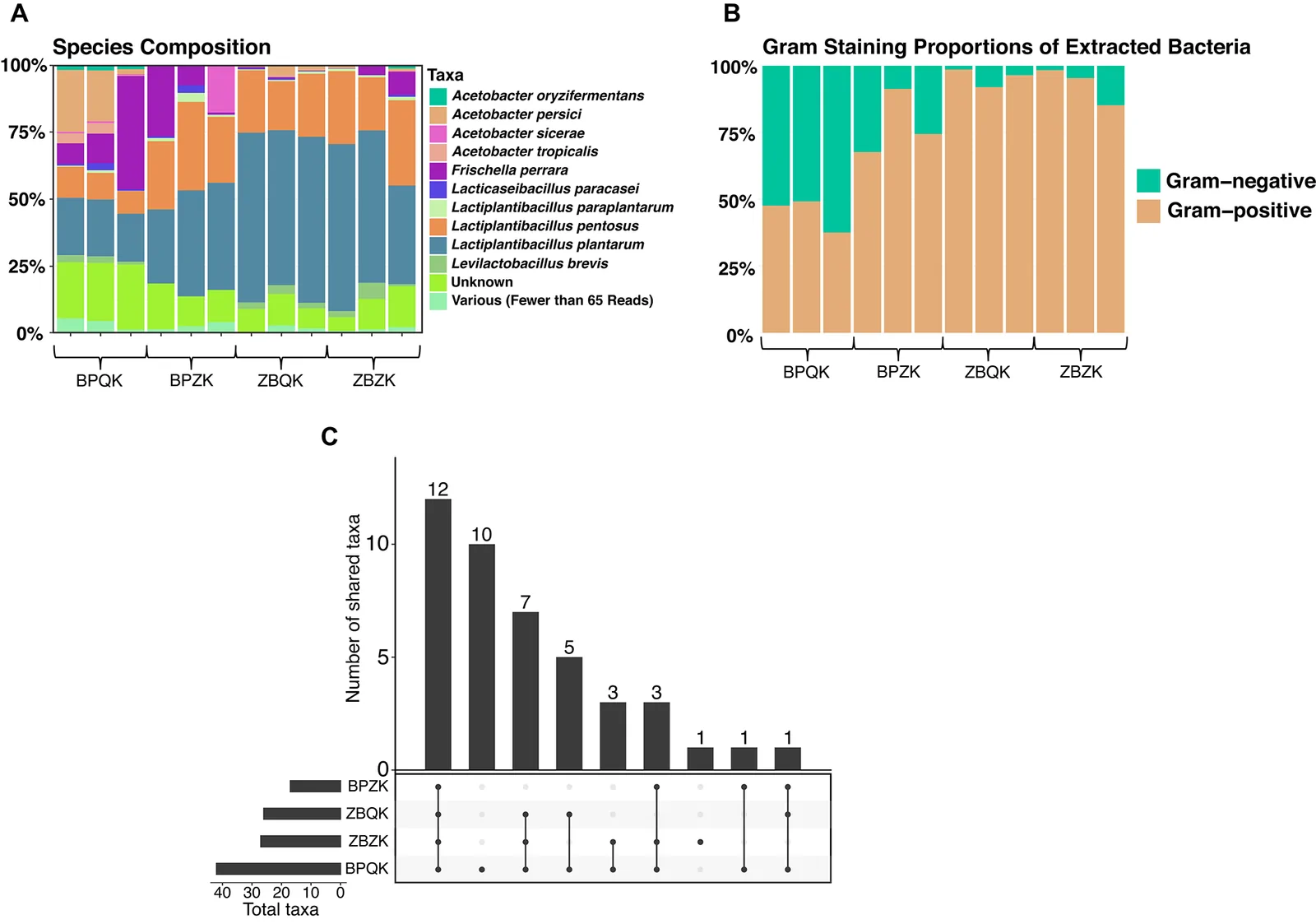
Taxonomic composition and Gram-stain distribution of *Drosophila melanogaster* gut microbiome samples across four DNA extraction protocols. **(A)** Relative abundance of bacterial species identified in gut samples processed using each extraction protocol. **(B)** Proportion of Gram-positive and Gram-negative bacterial species detected per protocol. **(C)** UpSet plot showing the overlap and unique distribution of bacterial species detected across the four DNA extraction protocols. Each bar represents the number of species shared among the indicated combination of protocols (connected dots), while single dots indicate taxa uniquely detected by a given protocol.

We first compared species composition across protocols (Fig. 3A). Taxonomic classification revealed bacterial genera such as *Lactobacillus* and *Acetobacter*, including species previously reported as part of the *Drosophila* commensal microbiota (reviewed in ^26,30,31^), such as *Lactiplantibacillus plantarum* (formerly *Lactobacillus plantarum*)*, Levilactobacillus brevis* (previously known as *Lactobacillus brevis),* and *Acetobacter tropicalis.* Our data suggest that our Canton-S wild-type fly line also contained additional *Lactobacillus* and *Acetobacter* species, including *A. persici*, *Lactiplantibacillus paraplantarum*, *Lactiplantibacillus pentosus*, and *Lacticaseibacillus paracasei,* which were detected at > 65 reads per sample. The relative abundance of these species varied among extraction protocols, with some samples also containing the anaerobic Gram-negative gammaproteobacterium *Frischella perrara*, originally described in the honeybee *Apis mellifera* ^47^ (Fig. 3A). Replicate samples within each protocol showed generally consistent community profiles, though BPQK and BPZK displayed greater variability in the relative abundance of *Acetobacter* and *Lactobacillus* species. A small fraction of reads (< 5%) were classified as “unknown” or represented low-abundance taxa (< 65 reads).

To confirm reliability of the taxonomic assignments, p-distances among reference sequences were calculated in MEGA12, and the relationships among detected species were visualized using Neighbor-Joining phylogenetic tree (**Supplementary Fig. 4**).

Gram-staining classification of the identified taxa indicated that the BPQK protocol extracted both Gram-positive and Gram-negative bacteria in nearly equal proportions (Fig. 3B). The BPZK protocol also recovered representatives of both groups, though Gram-positive species were slightly more abundant. In contrast, the ZBQK and ZBZK protocols extracted fewer Gram-negative taxa, showing a clear bias toward Gram-positive bacteria.

To compare overlap among detected taxa, an UpSet plot was constructed (Fig. 3C). Twelve bacterial species (excluding unknowns) were shared among all protocols and included *F. perrara* and *L. plantarum*. However, only the BPQK protocol successfully recovered gDNA for all taxa detected across methods, with the exception of *Levilactobacillus suantsaiihabitans*, which was uniquely identified in the ZBZK protocol. In addition, BPQK uniquely detected *Frischella japonica* and *Listeria cossartiae*, whereas ZBZK exclusively recovered *Levilactobacillus suantsaiihabitans* (**Supplementary Fig. 5**).

### *Drosophila* gut microbiota samples extracted with the BPQK protocol were associated with broader taxonomic recovery and higher alpha diversity

**Fig. 4:**
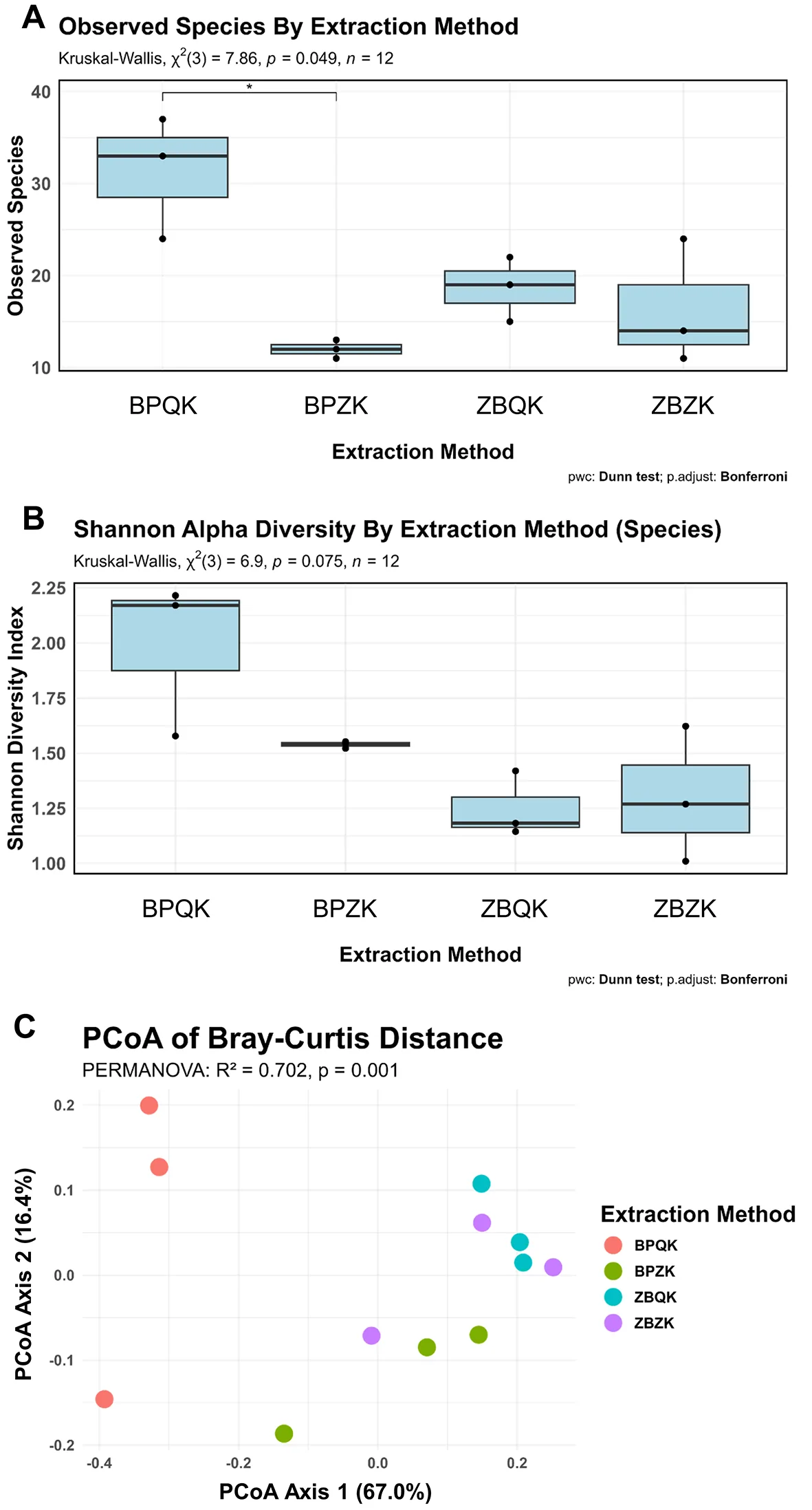
Alpha and beta diversity of *Drosophila melanogaster* gut microbiome samples across DNA extraction protocols. **(A)** Observed species richness across extraction protocols. **(B)** Shannon diversity index values for each protocol. **(C)** Principal Coordinates Analysis (PCoA) of Bray–Curtis dissimilarities showing sample clustering by extraction protocol. The percentage of variance explained by each PCoA axis is indicated in the figure.

We analyzed alpha and beta diversity metrics from the gut bacteria datasets obtained across the four extraction protocols (Fig. 4). Among all protocols, BPQK samples displayed the highest observed species richness, with a median of approximately 33 species (Kruskal–Wallis, χ2(3) = 7.86, p = 0.049; Bonferroni-corrected post-hoc tests significant), whereas the remaining protocols showed medians below 20 (Fig. 4A). Consistent with these findings, Shannon diversity indices showed a higher, though not significant, trend for BPQK compared to the other protocols (Fig. 4B). To confirm that the higher number of observed species captured by the BPQK extraction method is not a result of higher sequencing depth, we applied a relative abundance threshold filtering of 0.1%. While no significant differences across extraction protocols were observed when unknowns were included in the analysis (**Supplementary Fig. 6A**), significant differences emerged after filtering this category (**Supplementary Fig. 6B**; Kruskal–Wallis, χ2(3) = 8.27, p = 0.041). Post-hoc Dunn tests with Bonferroni correction identified a significantly higher number of observed species between BPQK and BPZK (adjusted p = 0.033). This increased richness was interpreted as broader taxonomic recovery rather than as a standalone indicator of extraction accuracy.

Principal Coordinates Analysis (PCoA) based on Bray–Curtis dissimilarities (using Bray–Curtis dissimilarity ~ group as the model, as described above) revealed modest overall clustering patterns by extraction method (Fig. 4C). PERMANOVA confirmed a significant effect of the group (i.e., DNA extraction and tissue homogenization method) on community composition (R2 = 0.702, p = 0.001), with BPQK samples forming a cluster distinct from the rest of the protocols. To ensure these results were not driven by unequal dispersion, we tested the homogeneity of multivariate dispersion; distances to group centroids did not differ significantly among methods for the *Drosophila* gut dataset (ANOVA: F = 0.906, p = 0.4796; **Supplementary Fig. 3B**), indicating comparable variance within groups. To determine if the observed differences were attributable to the DNA extraction or tissue homogenization methods, we performed an additional factorial PERMANOVA, as described above. DNA extraction method explained a smaller proportion of the variation (R2 = 0.148) and was not statistically significant (p = 0.087). In contrast, the tissue homogenization method significantly influenced microbial community composition (PERMANOVA, R2 = 0.377, p = 0.005).

To further assess potential sequencing run (batch) effects, aliquots of selected *D. melanogaster* gut DNA extracts were sequenced independently across two Oxford Nanopore sequencing runs (selected samples are indicated in Table 1). After excluding taxa representing < 0.1% relative abundance in both runs, duplicates showed significant positive correlations in species-level relative abundance profiles between sequencing runs (Spearman’s ρ = 0.6–0.89, *P* < 0.01; Pearson’s *r* = 0.94–0.99, *P* < 0.01), supporting the overall reproducibility of the sequencing workflow despite the expected technical variability associated with Nanopore sequencing.

## Discussion

This study evaluated various DNA extraction methods and tissue lysis protocols to determine their effectiveness in profiling the gut microbiome of *D. melanogaster,* using an MCS as a control. Our overarching goal was to develop a robust and reproducible workflow that combines commercially available extraction kits with a user-friendly bioinformatic pipeline, facilitating implementation in laboratories with diverse technical and computational resources. Our results demonstrate that the choice of tissue homogenization and DNA extraction method significantly influences microbial diversity and composition in both *D. melanogaster* gut samples and an MCS, with distinct impacts on species detection, alpha diversity, and beta diversity. We also found that sequencing and taxonomic classification contribute to the observed differences, particularly at the species level, underscoring the importance of interpreting species-level diversity estimates with caution.

Diversity metrics were interpreted cautiously in the MCS, as higher richness may reflect sequencing or classification artifacts rather than improved extraction performance. Therefore, protocol performance in the MCS was evaluated primarily based on recovery accuracy of expected taxa. Our findings reveal protocol-specific tradeoffs, including differences in the recovery of Gram-positive versus Gram-negative bacteria. The BPQK protocol produced the most balanced representation of both groups in both MCS, and more notably, the *Drosophila* gut samples. Similar biases in estimates of communities due to extraction protocols have been reported for human stool samples^18^, or synthetic communities of human-associated bacteria^19^.

The composition of the MCS revealed discrepancies between the expected and observed microbial profiles. The detection of additional genera not included in the MCS could reflect either potential contamination, such as the ‘kitome’ (bacteria originating from extraction kits), sequencing artifacts, or assignment misclassifications^20^. To control for contamination, we sequenced an NTC which consisted of adding sterile, nuclease-free water to the first PCR reaction instead of gDNA. This control was processed alongside all test samples throughout the sequencing process. The NTC contained only two low-abundance bacterial taxa, *C. acnes* (formerly *Propionibacterium acnes*), a common human-associated bacterium, and a single environmental taxon (*R. saccharophilus*), neither of which was detected in the MCS or *Drosophila* gut samples. Moreover, we did not detect any taxa entirely unexpected for the MCS or *Drosophila* datasets, such as typical human skin or environmental contaminants. This suggests that no obvious reagent-derived contaminants were detected in our datasets, although low-level background contamination cannot be completely excluded. In addition, only two species (*Enterococcus faecalis* and *Limosilactobacillus fermentum*) were shared between the filtered MCS dataset and the *Drosophila* gut samples, and these were detected in the gut dataset at extremely low abundances (2 reads each, in only sample BPQK_1). In contrast, these taxa appeared at substantially higher counts in the MCS dataset, suggesting that these detections likely represent low-level taxonomic misclassification or background signal rather than true biological overlap. However, a full kitome control, which would include reagent-only extractions processed in parallel with the samples, remains warranted for future studies to comprehensively rule out reagent-derived contaminants.

To further assess the contribution of sequencing errors, we analyzed the false-positive taxa detected in the unfiltered MCS dataset using phylogenetic reconstruction. Most false positives clustered within the same genera as their corresponding true positives, with pairwise sequence identities exceeding 99% in most cases. This high sequence similarity suggests that these discrepancies likely result from basecalling inaccuracies associated with the ~ 1–3% error rate reported for Oxford Nanopore sequencing^48,49^, rather than from true species-level differences. These findings emphasize the need for caution when interpreting species-level classifications from full-length 16S rRNA data and highlight the value of confirmatory sequencing using complementary platforms.

Interestingly, even the BPQK method, which detected 7 out of the 8 expected species in the MCS, did not enable the identification of *Listeria monocytogenes* at the species level, although *Listeria* was identified at the genus level. This Gram-positive bacterium is known for its thick, teichoic-acid-rich peptidoglycan cell wall^50^ but should, in principle, be easier to lyse than the spore-forming *Bacillus subtilis*^51^, which was successfully detected by all protocols. This suggests that *L. monocytogenes* sequences may cluster with other *Listeria* species due to their high 16S rRNA gene sequence similarity. Indeed, in the unfiltered MCS dataset, at least nine *Listeria* species were identified, with *Listeria cossartiae* detected in all samples and showing the highest relative abundance among the genus. Phylogenetic analysis using MEGA12 confirmed that the 16S gene sequence of *L. cossartiae* shares > 99% identity with *L. marthii, L. swaminathanii, L. innocua*, and *L. welshimeri*, and ~ 98% with *L. monocytogenes* (BLASTn), supporting the likelihood of misassignment at the species level when using 16S data alone.

The absence of *E. coli,* together with *B. subtilis* in some protocols, despite *E. coli* being easily lysed^51^, suggests that differences in DNA recovery may not stem solely from incomplete lysis but potentially from premature lysis and subsequent DNA degradation in more fragile species. Similarly, previous studies using defined microbial communities have reported that *E. coli* may be underrepresented despite its known presence, indicating that DNA recovery can vary depending on extraction conditions and community composition^19^. Together, these findings underscore the importance of balancing enzymatic and mechanical disruption steps to avoid compromising genomic DNA integrity during extraction.

Species-level analysis of *Drosophila* gut samples further highlighted the variability among lysis/extraction protocols. The BPQK protocol consistently recovered *L. plantarum*, *A. percici*, and *F. perrara*, suggesting broad sensitivity for both Gram-negative and Gram-positive taxa. In contrast, BPZK showed high abundances of *L. plantarum* and *L. pentosus*, but reduced *Acetobacter* spp., while the Zymo bead-based protocols (ZBQK and ZBZK) primarily detected *L. plantarum* and *L. pentosus*, yet displayed a reduced detection of *F. perrara*. Together, these results demonstrate that the extraction methods directly shape the resulting taxonomic profile and involve trade-offs between taxonomic breadth and assignment accuracy. Although BPQK recovered the broadest representation of the fly gut community including both Gram-negative (*Acetobacter*) and Gram-positive (*Lactiplantibacillus*) bacteria, the MCS analyses indicate that this broader recovery was accompanied by lower precision due to a greater number of false-positive assignments.

Alpha diversity measures also differed significantly among protocols. In the MCS dataset, protocol performance was best evaluated by agreement with the expected composition rather than absolute richness. After filtering overclassified taxa, BPQK recovered the highest number of true positives but still showed compositional deviation from the expected equal-abundance standard. In contrast, in *Drosophila* gut samples, where true community structure is unknown, higher richness likely reflects broader taxon recovery rather than inflation alone. Therefore, protocol performance must be interpreted within the biological context of the sample type.

In the MCS, all protocols overestimated diversity relative to the expected, but after filtering, BPQK recovered the highest number of true-positive taxa, although compositional deviations from the expected equal-abundance standard remained. These results also highlight a trade-off between sensitivity and specificity among extraction protocols. Methods such as BPQK recovered a greater proportion of expected taxa (7 of 8 species), indicating higher sensitivity, but also produced additional species-level assignments likely reflecting taxonomic overclassification. In contrast, protocols such as ZBZK yielded fewer unexpected taxa but failed to recover several expected members of the mock community. Because taxa lost during DNA extraction cannot be recovered through downstream bioinformatic processing, workflows that maximize recovery of expected taxa while maintaining balanced Gram-positive and Gram-negative detection may provide a more informative representation of microbial communities.

In the *Drosophila* gut samples, BPQK displayed the highest observed richness and Shannon diversity, capturing a broader range of taxa than the other methods. BPZK showed moderately high diversity but fewer observed species, while both Zymo-bead based protocols yielded lower and more uniform values. These findings suggest that, while Zymo kits perform well in MCS, the Qiagen-based BPQK protocol is more effective for complex biological samples, such as fly gut tissue. These findings also highlight that diversity metrics should be interpreted alongside taxonomic accuracy and compositional structure when evaluating extraction performance.

Beta diversity analyses reinforced these observations. In both datasets, Bray–Curtis dissimilarity revealed clear protocol-dependent clustering, with BPQK samples forming distinct groups. In the *Drosophila* gut samples, the BPQK also clustered separately, consistent with its broader taxonomic recovery and reproducible community structure.

The observed differences in microbial composition, richness, and clustering patterns underscore the critical role of DNA extraction methods in shaping microbiome data. Protocols that effectively lyse both Gram-positive and Gram-negative bacteria, such as BPQK, are essential for comprehensive microbial profiling. In contrast, the Gram-positive bias observed in Zymo-bead based protocols limits detection of key taxa such as *Acetobacter*. These protocol-specific differences highlight that small variations in enzymatic or mechanical lysis can have substantial effects on apparent diversity and abundance. Because mechanical disruption and extraction chemistry were applied together within each commercial workflow, the relative contributions of homogenization method and lysis buffer composition could not be independently disentangled in this study. Together, the MCS and gut microbiome analyses demonstrate that discrepancies in detected taxa can arise from both the lysis/extraction method and sequencing artifacts, rather than from true biological variation among studies. Collectively, these findings underscore the importance of rigorous quality control, as reliable extraction and sequencing protocols should recover all expected taxa while minimizing false positives. To fully capture bacterial diversity within a sample, combining or splitting samples for complementary extraction methods may enhance the recovery of both Gram-positive and Gram-negative taxa.

### Recommendations, limitations and future studies

Our results showed that each protocol possesses advantages and limitations, which we have summarized in Table 3. Consequently, the most appropriate extraction protocol depends on the biological question being addressed. For studies seeking comprehensive characterization of the *Drosophila* gut microbiota or balanced recovery of taxa such as *Acetobacter* and *Lactobacillus*, BPQK may provide the greatest benefit. In contrast, studies prioritizing conservative taxonomic assignments or working with different microbial communities may favor protocols that recover fewer unexpected taxa, even at the expense of reduced taxonomic breadth (Table 2).

**Table 3:** Summary of the strengths, limitations, and potential applications of the four tissue homogenization and DNA extraction methods evaluated in this study. The table highlights the trade-offs observed for each protocol based on mock community and *Drosophila melanogaster* gut microbiome analyses. Recommendations are intended as practical guidance for selecting an extraction workflow according to study objectives.

Protocol	Strengths	Limitations	Considerations
BPQK (Blue Pestle + Qiagen kit)	Highest recovery of expected taxa in MCS; balanced Gram-positive/Gram-negative recovery; highest richness in gut samples; broadest taxonomic recovery	Highest number of false positives in MCS; most unique taxa detected in gut samples; greater likelihood of recovering additional species-level assignments requiring cautious interpretation	Maximizes sensitivity and broad taxonomic recovery but requires cautious interpretation of species-level assignments
BPZK (Blue Pestle + Zymo kit)	Good recovery of expected taxa; fewer false positives than BPQK	Missed expected *Escherichia* in MCS; lower richness than BPQK	Intermediate balance between sensitivity and specificity
ZBQK (Zymo Beads + Qiagen kit)	Moderate recovery of expected taxa; lower false positives than BPQK	Reduced Gram-negative recovery in gut samples; lower diversity than BPQK	Moderate overall performance with reduced Gram-negative recovery
ZBZK (Zymo Beads + Zymo kit)	Highest precision (fewest false positives) in the MCS	Lowest recall; failed to recover multiple expected taxa; strongest Gram-positive bias; lowest richness	Most conservative protocol but likely underestimates community diversity

Although this study provides valuable insights into the impact of DNA extraction methodology on microbiome composition using full-length 16S Nanopore sequencing, several limitations should be acknowledged. First, the number of biological samples analyzed was modest, which may limit the statistical power to detect subtle community differences, particularly given the multivariate nature of microbiome community analyses. However, this work prioritized methodological validation over population-level inference, and the consistent patterns observed across replicates support the reliability of the findings. Importantly, higher observed richness should not be interpreted as greater biological accuracy, as extraction methods may differentially recover DNA from specific taxa, thereby artificially altering diversity metrics independent of true community structure.

Second, while Oxford Nanopore Technologies (ONT) enables accessible full-length 16S sequencing, results were not cross-validated with a second sequencing platform such as PacBio HiFi. Future studies could strengthen taxonomic resolution and error correction by integrating hybrid sequencing approaches or by submitting extracted gDNA for parallel sequencing using alternative technologies. Although full-length 16S sequencing provides improved resolution, species-level classifications should still be interpreted cautiously, and confirmatory sequencing using alternative platforms is recommended. In addition, because extraction protocols were processed across separate sequencing runs, some contribution of run-to-run variability cannot be completely excluded.

Third, the ONT 16S Barcoding Kit used in this study provides only 24 unique barcodes, constraining throughput and necessitating two sequencing runs. This introduces potential batch effects; however, identical reagents, flow cell chemistry, and analysis parameters were applied across runs to minimize technical variation. Additionally, specific samples were sequenced in both runs as internal controls to assess consistency and detect potential run-to-run variability. The significant positive correlations observed between duplicated samples sequenced across independent Oxford Nanopore runs further support the reproducibility of the sequencing workflow and suggest that the protocol-dependent differences identified in this study were not solely attributable to run-to-run technical variability.

Fourth, primer design may also influence taxonomic representation. The universal 27F primer used in this study contained a “CC” at the degenerate positions, whereas alternative versions incorporating “YM” or “CM” degeneracies have been shown to improve amplification across diverse bacterial taxa. Such primer sequence differences could affect the detection of certain lineages, particularly those with mismatches at the 27F binding site. Future work could compare these primer variants to evaluate their impact on taxonomic coverage and relative abundance estimates.

Fifth, our taxonomic classification depended on the databases available within EPI2ME. Because our goal was to create a workflow accessible to laboratories with limited computing infrastructure, we used this cloud-based platform to avoid computationally intensive basecalling and alignment steps. However, the limited reference databases in EPI2ME resulted in several unclassified reads. This limitation can be mitigated by uploading custom-curated databases to EPI2ME, after which species abundance tables can be processed through the R pipeline provided in this study. This workflow provides a practical alternative to existing pipelines such as NanoCLUST^52^, NanoRTax^53^, and EMU^54^, which require access to high-performance computing infrastructure or paid cloud-based resources. Similarly, future work could explore more stringent quality score thresholds and confidence-based taxonomic filtering strategies to further reduce species-level overclassification resulting from specific annotation pipelines.

Additionally, because microbial communities differ substantially across host systems, researchers may benefit from developing mock community standards tailored to the expected taxa in their specific biological model. For models such as *D. melanogaster*, where core commensal taxa are relatively well defined and many are culturable, constructing a customized microbial standard could enable more precise benchmarking of extraction efficiency and taxonomic assignment accuracy. Such model-specific validation would further strengthen confidence in microbiome profiling workflows. These observations highlight that, while commercial mock communities are useful for benchmarking extraction workflows, model-specific standards composed of taxa commonly found in the biological system under study may provide more informative validation.

Sixth, while this work focused on qualitative comparisons, future studies incorporating quantitative metrics, including DNA yield, integrity, and lysis efficiency, could help clarify the mechanisms driving extraction bias. In mixed microbial communities containing both easily lysed and more resilient organisms, premature lysis or excessive mechanical disruption may compromise DNA integrity in fragile taxa. Enzymatic digestion time may also influence Gram-specific recovery. In preliminary experiments conducted in our laboratory, shorter Proteinase K incubations (approximately 3 h) resulted in strong detection of Gram-negative taxa but reduced recovery of Gram-positive organisms. For this reason, and following the protocol described by Bombin et al. (2020)^55^, we implemented an overnight Proteinase K digestion step in the Qiagen workflow to promote more balanced lysis across bacterial groups. Nonetheless, further work is needed to systematically evaluate how Proteinase K incubation time influences Gram-positive and Gram-negative recovery. Additionally, future studies should determine whether the high abundance of *Bacillus* observed in BPQK samples reflects genuine recovery of spore-forming bacteria or overrepresentation due to differential DNA retention or purification efficiency.

Despite these limitations, the experimental design successfully demonstrates clear protocol-dependent biases during DNA extraction and provides a framework for establishing standardized, reproducible workflows for ONT-based microbiome studies, particularly in low-resource or teaching laboratory environments.

## Conclusions

This study demonstrates that DNA extraction protocols exert a major influence on measures of microbial diversity and composition in *Drosophila melanogaster* gut microbiome studies. Among the approaches tested, Blue Pestle homogenization combined with the modified Qiagen DNeasy Blood & Tissue Kit (BPQK) provided balanced recovery of Gram-positive and Gram-negative taxa and was associated with broader taxonomic recovery in *Drosophila* gut samples. Nonetheless, all protocols introduced method-specific biases that affected microbial profiles, emphasizing the need for careful method selection and validation in microbiome research. Moving forward, the development and adoption of standardized, user-friendly workflows, using commercially available kits and accessible bioinformatic tools, will be essential to enhance accuracy, reproducibility, and cross-study comparability in long-read microbiome analyses.

Protocols for full-length 16S rRNA sequencing should also be optimized for each tissue or sample type studied (e.g., *Drosophila* gut) and published in a standardized manner, similar to the approach of the Earth Microbiome Project, which established reference protocols for 16S amplicon sequencing across diverse sample types such as feces, oral, skin, soil, and water^56,57^. This is particularly important as ongoing efforts aim to develop a roadmap for establishing guidelines that promote equitable sequence data reuse and interoperability in public microbiome datasets, which should also include detailed documentation of the genomic DNA extraction protocols used^58^.

## Materials & Methods

### *Drosophila melanogaster* husbandry and gut microbiome sample preparation

Wild-type *Drosophila melanogaster* Canton-S flies were raised in a 25℃ incubator with 12 h:12 h light:dark cycle with ~ 60% humidity. Newly eclosed female flies were collected, mated and aged for five days. All progeny used in this study consisted of a pooled cohort derived from 6–8 bottles. Following pooling, flies were randomly selected to generate three replicate gut pools for each DNA extraction protocol, with each pool consisting of 10 female flies (30 flies per protocol). All replicate samples were prepared simultaneously on the same day prior to gut dissections. Flies were then dissected. Prior to dissections, flies were surface-sterilized by washing in 70% ethanol for 30 s followed by two washes in sterile 1 × phosphate-buffered saline (PBS) (Fisher Cat. No. BP399500). Guts were dissected under flame in drops of sterile 1 × PBS on a sterile Petri dish. Crops and Malpighian tubules were removed from each gut before pooling in 500 µL of sterile PBS on ice. After dissections and pooling (10 guts per sample), PBS was removed, leaving only the tissues. During dissection, samples were maintained in a 1.5 mL Eppendorf tube cooling rack pre-chilled at -20ºC to prevent thawing and were subsequently stored at − 80 °C until processing.

### Microbial mock community standard

The microbial MCS, ZymoBIOMICS Microbial Community Standard (Cat. No. D6300, Zymo Research) was obtained from Zymo Research (Irvine, CA) and was included in the present study as a control. The MCS contains cells of five Gram-positive bacteria (i.e., *Lactobacillus fermentum, Enterococcus faecalis, Staphylococcus aureus, Listeria monocytogenes,* and *Bacillus subtilis*), three Gram-negative bacteria (i.e., *Pseudomonas aeruginosa, Escherichia coli,* and *Salmonella enterica*), and two yeast species (i.e., *Saccharomyces cerevisiae* and *Cryptococcus neoformans*). Each bacterial species is present at an equal genomic DNA proportion (12%), while the two yeast species represent 2% each of the total genomic DNA.

### Tissue homogenization and bacterial genomic DNA extraction

Two distinct tissue lysis methods were tested: blue pestle homogenization and Zymo bead-beating: i) For blue pestle homogenization, frozen guts samples (stored at − 80 °C as described above) or microbial community standard (MCS) samples were thawed on ice and mixed with the lysis buffer provided in the respective extraction kit, following the manufacturer’s protocol. The samples were then homogenized using a hand-held homogenizer (VWR® Disposable Pellet Mixers and Cordless Motor, Cat. No. 47747–370; VWR International) for approximately 3 min. For gut samples, the homogenization time was determined empirically, ending when no large tissue fragments were visible by direct inspection.

ii) For bead beating homogenization, gut or MCS samples were first combined with the lysis buffer from the indicated kit and transferred to ZymoBIOMICS™ BashingBeads Lysis Tubes (2 mm). The tubes were processed in a Vortex Genie 2 (Model G-560, Scientific Industries Inc.) equipped with an adapter containing a Horizontal Microtube Holder (SI-H524, Scientific Industries Inc.) for 20 min at maximum speed (3,200 rpm).

Following homogenization, genomic DNA was extracted using two commercial kits: i) ZymoBIOMICS™ DNA Miniprep Kit (Cat. No. D4300, Zymo Research) and the ii) Qiagen DNeasy Blood & Tissue Kit (Cat. No. 69504, Qiagen). The ZymoBIOMICS™ DNA Miniprep Kit protocol was followed without modification. For the Qiagen kit, the Proteinase K digestion step was extended overnight at 56ºC according to the procedure described by Bombin et al*.* (2020)^55^. The MCS samples were processed simultaneously with gut samples.

DNA yields for the *Drosophila* samples were quantified following extraction from pools of 10 fruit fly guts (3 replicates per protocol). Total DNA yields varied depending on the extraction protocol, ranging from 0.1 µg to 6.2 µg per sample (1–62 ng/µL in 100 µL elution volume). Specifically, BPQK samples yielded 5.9–6.2 µg, ZBQK 1.1–3.2 µg, BPZK 1.1–3.3 µg, and ZBZK 0.1–2.6 µg of total DNA.

This experimental design resulted in four treatment groups: Blue Pestles + Qiagen Kit (BPQK), Zymo Beads + Qiagen Kit (ZBQK), Blue Pestles + Zymo Kit (BPZK), and Zymo Beads + Zymo Kit (ZBZK) (Table 1). Each protocol was applied to both the MCS (n = 4 replicates) and *D. melanogaster* gut samples (n = 3 replicates, 10 guts per replicate). Sequencing was performed in two independent runs, with BPQK and ZBQK processed in Run 1, and BPZK and ZBZK in Run 2. Some selected samples were sequenced in both runs, as indicated in Table 1, to assess potential batch effects.

### Full-length 16S rRNA gene amplification

Full-length 16 s rRNA genes were amplified by PCR using LongAmp® Taq DNA Polymerase (Cat. No. M0533S, New England BioLabs), and the universal bacterial primers 27F (5′-AGA GTT TGA TCC TGG CTC AG-3′) (Integrated DNA Technologies; Cat. No. 51–01-19–06) and 1492R (5′-ACG GCT ACC TTG TTA CGA CTT-3′) (Integrated DNA Technologies; Cat. No. 51–01-19–07). These primers amplify approximately 1500 bp of the 16S rRNA gene, allowing full-length 16S rRNA sequencing. Prior to amplification, DNA concentrations were normalized so that 10 ng of template DNA was used per PCR reaction.

PCR conditions consisted of an initial denaturation at 94ºC for 2 min, followed by 34 cycles 30 s, 58 °C for 30 s, and 68 °C for 90 s, with a final extension of 68 °C for 90 s. PCR products were verified by agarose gel electrophoresis and purified using the Gene Choice PCR Purification Kit (Cat. No. 96–302, Genesee Scientific), according to the manufacturer’s instructions DNA quantity and purity were assessed with a Nanodrop spectrophotometer.

A non-template control (NTC) containing nuclease-free sterile water instead of genomic DNA was included in each PCR run. The NTC was processed identically to experimental samples through all subsequent steps, including sequencing, to serve as a control for contamination.

### Library preparation and 16 s rRNA sequencing

Library preparation was performed using the Nanopore 16S Barcoding Kit 24 V14 (Cat. No. SQK-16S114.24, Oxford Nanopore), following the manufacturer’s Rapid Sequencing DNA protocol (Version 16S_9199_v114_revE_11Dec2024, Oxford Nanopore). Due to the kit’s limitation of 24 unique barcodes, two sequencing runs were required (as indicated in Table 1). To assess and partially control for potential batch effects, aliquots of the same extracted DNA samples were included in both sequencing runs under distinct barcodes, enabling cross-run comparison of identical samples. The NTC control was also sequenced in both runs.

Barcodes were attached to amplicons by PCR according to the manufacturer’s instructions. Barcoded libraries were quantified using a Qubit fluorometer (Thermo Fisher Scientific) and pooled in equimolar ratios. The pooled libraries were purified with AMPure XP Beads (Beckman Coulter) using 80% ethanol prepared in nuclease-free water, and DNA was eluted in 10 µL of nuclease-free water. Final library concentrations were measured using the Qubit fluorometer, and 50 femtomoles of DNA were loaded for sequencing.

Sequencing was carried out on a GridION platform (Oxford Nanopore Technologies using R10.4.1 MinION flow cells; Cat. No. FLO-MIN114, Oxford Nanopore) operated under the high-fidelity (high-accuracy) sequencing mode to minimize error rates.

### Taxonomic classification and diversity calculations

Sequencing data were analyzed using the 16S workflow (v1.5.0) in Oxford Nanopore’s cloud-based EPI2ME platform (https://epi2me.nanoporetech.com/), which includes the following steps: high-accuracy basecalling (using Dorado version 7.8.3 for run 1 and Dorado version 7.9.8 for run 2), demultiplexing, barcode trimming, read filtering and alignment to reference database. Taxonomic classification was performed with Minimap2 (version 2.28-r1209) against the NCBI 16S and 18S rRNA database (ONT given name: ncbi_targeted_loci_16s_18s.fna). Only reads between 1200 and 1800 base pairs and with a minimum quality score of 18 were retained for downstream analysis.

For diversity calculations and data visualization, the species abundance table generated by EPI2ME (**Supplementary Data 1**) was exported and processed in R (version 4.4.2) and RStudio (v2025.09.0 + 387) and a custom analysis script. All analyses were performed on a Beelink SER5 MAX Mini PC equipped with an AMD Ryzen 7 6800U processor (8 cores, 16 threads, up to 4.7 GHz), 32 GB RAM, and a 500 GB PCIe 4.0 SSD running Windows 11 Pro.

Rarefaction was performed to standardize sequencing depth (962 sequences for MCS and 1,108 sequences for gut samples) (**Supplementary Fig. 7**). Alpha diversity metrics (including observed species richness and Shannon diversity index) were computed with the vegan package (v2.7–1). After the Kruskal–Wallis test, pairwise group comparisons were performed using the Dunn test with Bonferroni-adjusted p-values. Beta diversity was assessed using Bray–Curtis dissimilarities, and significance among extraction methods was tested by PERMANOVA computed with the vegan package (v2.7–1) and the adonis2 function. To further determine the contributions of tissue homogenization and DNA extraction method, an additional factorial PERMANOVA was performed using the model Bray–Curtis dissimilarity ~ tissue homogenization method + DNA extraction method. The expected mock community profile was excluded from this factorial analysis since it did not undergo tissue homogenization or DNA extraction. Marginal effects of each factor were evaluated using the by = “margin” option with 9,999 permutations. Pairwise PERMANOVA comparisons were performed to identify differences between specific extraction groups, and p-values were adjusted for multiple comparisons using the False Discovery Rate (FDR) correction. To assess whether differences in community composition could be attributed to unequal variability among groups, homogeneity of multivariate dispersion was tested using the betadisper function from the vegan package (v2.7–1), and statistical significance was evaluated using ANOVA and the TukeyHSD tests. All analyses were conducted in RStudio (version 2025.09.2 + 418).

To assess potential sequencing run (batch) the reproducibility of Nanopore sequencing across independent sequencing runs, and evaluate potential sequencing run (batch) effects, aliquots of selected *D. melanogaster* gut DNA extracts were sequenced in two separate runs under different barcode assignments. Species-level read counts were converted to relative abundances, and taxa representing < 0.1% relative abundance in both sequencing runs were excluded to minimize the influence of low-abundance detections. Concordance between sequencing runs was evaluated using both Spearman’s rank correlation (ρ) and Pearson’s correlation coefficient (*r*) based on species-level relative abundance profiles.

Phylogenetic tree construction and UniFrac distance calculations (data not shown) were performed using the phyloseq package (v1.50.0). Gram-stain assignments were generated with the AMR package (v3.0.0), and data visualization was completed with ggplot2 (v4.0.0). Following rarefaction of the species count matrix to the minimum sequencing depth using the rrarefy function in the vegan R package, read counts were converted to presence/absence values. Presence and overlap of detected species across extraction methods were visualized using an UpSet plot. Replicate samples for each extraction method were collapsed such that a species was considered present if detected in at least one replicate. A binary matrix representing species presence across extraction methods (BPQK, BPZK, ZBQK, and ZBZK) was generated and used to visualize shared and unique taxa among extraction methods using the UpSetR R package.

Venn diagrams were produced with ggvenn (v0.1.10).

### Evolutionary relationships of taxa

Neighbor-joining trees for both MCS and *Drosophila* gut bacteria samples were constructed in MEGA12 (v12.0.14) ^59^. Briefly, the evolutionary history was inferred using the Neighbor-Joining method ^60^, and the robustness of the tree topology was assessed using bootstrap analysis with 1,000 replicates ^61^. The optimal tree for the MCS dataset had a total branch length of 0.908, and the tree for the gut samples had a total branch length of 0.931 (**Supplementary Fig. 2 and 4).** Evolutionary distances were calculated using the p-distance method ^62^ and are expressed as the number of base differences per site. Bootstrap support values (percentages) are shown next to each internal node. The analyses included 111 nucleotide sequences for the MCS and 82 sequences for the gut samples (**Supplementary Fig. 2 and 4**). Ambiguous positions were handled using the pairwise deletion option, resulting in a final dataset comprising 1,652 positions for MCS and 1,613 positions for gut samples.

## Supplementary Information

Supplementary Information 1.

Supplementary Information 2.

Supplementary Information 3.

## Data Availability

Raw sequencing data are available at the NCBI Sequence Archive (SRA) under the BioProject accession number PRJNA1367163.

## Code availability

The custom script for downstream analysis of full-length 16S rRNAseq data generated from EPI2ME abundance tables is available on GitHub (https://github.com/carlosqui12/Scientific_Reports_2026). Processed abundance tables are provided in **Supplementary Data 1**.

## References

[1] Sekirov, I., Russell, S. L., Caetano, L., Antunes, M. & Finlay, B. B. Gut Microbiota in *Health and Disease*. 10.1152/physrev.00045.2009.-Gut (2010) 10.1152/physrev.00045.2009.-Gut. 20664075

[2] Turnbaugh, P. J. et al. The Human Microbiome Project. *Nature* **449**, 804–810 (2007). 10.1038/nature06244 PMC3709439 17943116

[3] Hou, K. et al. Microbiota in health and diseases. *Signal Transduct. Target. Ther.* 10.1038/s41392-022-00974-4 (2022). PMC9034083 35461318

[4] Afzaal, M. et al. Human gut microbiota in health and disease: Unveiling the relationship. *Front. Microbiol.* 10.3389/fmicb.2022.999001 (2022). PMC9549250 36225386

[5] Allaband, C. et al. Microbiome 101: Studying, analyzing, and interpreting gut microbiome data for clinicians. *Clin. Gastroenterol. Hepatol.* **17**, 218–230 (2019). 10.1016/j.cgh.2018.09.017 PMC6391518 30240894

[6] Ranjan, R., Rani, A., Metwally, A., McGee, H. S. & Perkins, D. L. Analysis of the microbiome: Advantages of whole genome shotgun versus 16S amplicon sequencing. *Biochem. Biophys. Res. Commun.* **469**, 967–977 (2016). 10.1016/j.bbrc.2015.12.083 PMC4830092 26718401

[7] Ursell, L. K., Metcalf, J. L., Parfrey, L. W. & Knight, R. Defining the human microbiome. *Nutr. Rev.* **70**, S38–S44 (2012). 10.1111/j.1753-4887.2012.00493.x PMC3426293 22861806

[8] Johnson, J. S. et al. Evaluation of 16S rRNA gene sequencing for species and strain-level microbiome analysis. *Nat. Commun.* **10**, 5029 (2019). 10.1038/s41467-019-13036-1 PMC6834636 31695033

[9] Clarridge, J. E. Impact of 16S rRNA gene sequence analysis for identification of bacteria on clinical microbiology and infectious diseases. *Clin. Microbiol. Rev.* **17**, 840–862 (2004). 10.1128/CMR.17.4.840-862.2004 PMC523561 15489351

[10] Aja-Macaya, P. et al. Nanopore full length 16S rRNA gene sequencing increases species resolution in bacterial biomarker discovery. *Sci. Rep.* **15**, 26486 (2025). 10.1038/s41598-025-10999-8 PMC12280133 40691262

[11] Hu, T., Chitnis, N., Monos, D. & Dinh, A. Next-generation sequencing technologies: An overview. *Hum. Immunol.* **82**, 801–811 (2021). 10.1016/j.humimm.2021.02.012 33745759

[12] Wang, Y., Zhao, Y., Bollas, A., Wang, Y. & Au, K. F. Nanopore sequencing technology, bioinformatics and applications. *Nat. Biotechnol.* **39**, 1348–1365 (2021). 10.1038/s41587-021-01108-x PMC8988251 34750572

[13] Zheng, P. et al. Nanopore sequencing technology and its applications. *MedComm* **4**, e316 (2023). 10.1002/mco2.316 PMC10333861 37441463

[14] Szoboszlay, M. et al. Nanopore is preferable over Illumina for 16S amplicon sequencing of the gut microbiota when species-level taxonomic classification, accurate estimation of richness, or focus on rare taxa is required. *Microorganisms*10.3390/microorganisms11030804 (2023). PMC10059749 36985377

[15] Karst, S. M. et al. High-accuracy long-read amplicon sequences using unique molecular identifiers with Nanopore or PacBio sequencing. *Nat. Methods* **18**, 165–169 (2021). 10.1038/s41592-020-01041-y 33432244

[16] Butler, I. et al. Standardization of 16S rRNA gene sequencing using nanopore long read sequencing technology for clinical diagnosis of culture negative infections. *Front. Cell. Infect. Microbiol.* 10.3389/fcimb.2025.1517208 (2025). PMC11922894 40115075

[17] Sohrabi, M. et al. The yield and quality of cellular and bacterial DNA extracts from human oral rinse samples are variably affected by the cell lysis methodology. *J. Microbiol. Methods* **122**, 64–72 (2016). 10.1016/j.mimet.2016.01.013 26812577

[18] Costea, P. I. et al. Towards standards for human fecal sample processing in metagenomic studies. *Nat. Biotechnol.* **35**, 1069–1076 (2017). 10.1038/nbt.3960 28967887

[19] Yuan, S., Cohen, D. B., Ravel, J., Abdo, Z. & Forney, L. J. Evaluation of methods for the extraction and purification of DNA from the human microbiome. *PLoS ONE*10.1371/journal.pone.0033865 (2012). PMC3311548 22457796

[20] Salter, S. J. et al. Reagent and laboratory contamination can critically impact sequence-based microbiome analyses. *BMC Biol.* 10.1186/s12915-014-0087-z (2014). PMC4228153 25387460

[21] Felczykowska, A., Krajewska, A., Zielińska, S. & Łoś, J. M. Sampling, metadata and DNA extraction - important steps in metagenomic studies. *Acta Biochim. Pol.* **62**, 151–160 (2015). 10.18388/abp.2014_916 25680373

[22] Knight, R. et al. Best practices for analysing microbiomes. *Nat. Rev. Microbiol.* **16**, 410–422 (2018). 10.1038/s41579-018-0029-9 29795328

[23] Greathouse, K. L., Sinha, R. & Vogtmann, E. DNA extraction for human microbiome studies: The issue of standardization. *Genome Biol.* 10.1186/s13059-019-1843-8 (2019). PMC6802309 31639026

[24] Nearing, J. T., Comeau, A. M. & Langille, M. G. I. Identifying biases and their potential solutions in human microbiome studies. *Microbiome*10.1186/s40168-021-01059-0 (2021). PMC8132403 34006335

[25] Hermans, S. M., Buckley, H. L. & Lear, G. Optimal extraction methods for the simultaneous analysis of DNA from diverse organisms and sample types. *Mol. Ecol. Resour.* **18**, 557–569 (2018). 10.1111/1755-0998.12762 29394525

[26] Broderick, N. A. & Lemaitre, B. Gut-associated microbes of *Drosophila melanogaster*. *Gut Microbes* **3**, 307–321 (2012). 10.4161/gmic.19896 PMC3463489 22572876

[27] Douglas, A. E. Simple animal models for microbiome research. *Nat. Rev. Microbiol.* **17**, 764–775 (2019). 10.1038/s41579-019-0242-1 31417197

[28] Buchon, N., Broderick, N. A. & Lemaitre, B. Gut homeostasis in a microbial world: Insights from *Drosophila melanogaster*. *Nat. Rev. Microbiol.* **11**, 615–626 (2013). 10.1038/nrmicro3074 23893105

[29] Erkosar, B. & Leulier, F. Transient adult microbiota, gut homeostasis and longevity: Novel insights from the *Drosophila* model. *FEBS Lett.* **588**, 4250–4257 (2014). 10.1016/j.febslet.2014.06.041 24983497

[30] Wong, C. N. A., Ng, P. & Douglas, A. E. Low-diversity bacterial community in the gut of the fruitfly *Drosophila melanogaster*. *Environ. Microbiol.* **13**, 1889–1900 (2011). 10.1111/j.1462-2920.2011.02511.x PMC3495270 21631690

[31] Ludington, W. B. & Ja, W. W. *Drosophila* as a model for the gut microbiome. *PLoS Pathog.* **16**, e1008398 (2020). 10.1371/journal.ppat.1008398 PMC7179820 32324814

[32] Trinder, M., Daisley, B. A., Dube, J. S. & Reid, G. *Drosophila melanogaster* as a high-throughput model for host-microbiota interactions. *Front. Microbiol.* 10.3389/fmicb.2017.00751 (2017). PMC5408076 28503170

[33] Shin, S. C. et al. *Drosophila* microbiome modulates host developmental and metabolic homeostasis via insulin signaling. *Science* **334**, 670–674 (2011). 10.1126/science.1212782 22053049

[34] Chaston, J. M., Newell, P. D. & Douglas, A. E. Metagenome-wide association of microbial determinants of host phenotype in drosophila melanogaster. *mBio* **5**, (2014). 10.1128/mBio.01631-14 PMC4196228 25271286

[35] Chaston, J. M., Dobson, A. J., Newell, P. D. & Douglas, A. E. Host genetic control of the microbiota mediates the *Drosophila* nutritional phenotype. *Appl. Environ. Microbiol.* **82**, 671–679 (2016). 10.1128/AEM.03301-15 PMC4711117 26567306

[36] Wong, A. C. N., Dobson, A. J. & Douglas, A. E. Gut microbiota dictates the metabolic response of Drosophila to diet. *J. Exp. Biol.* **217**, 1894–1901 (2014). 10.1242/jeb.101725 PMC4037322 24577449

[37] Martino, M. E. et al. Bacterial adaptation to the host’s diet is a key evolutionary force shaping *Drosophila*-*Lactobacillus* symbiosis. *Cell Host Microbe* **24**, 109-119.e6 (2018). 10.1016/j.chom.2018.06.001 PMC6054917 30008290

[38] Erkosar, B. et al. Pathogen virulence impedes mutualist-mediated enhancement of host juvenile growth via inhibition of protein digestion. *Cell Host Microbe* **18**, 445–455 (2015). 10.1016/j.chom.2015.09.001 PMC4617634 26439865

[39] Storelli, G. et al. *Lactobacillus plantarum* promotes *Drosophila* systemic growth by modulating hormonal signals through TOR-dependent nutrient sensing. *Cell Metab.* **14**, 403–414 (2011). 10.1016/j.cmet.2011.07.012 21907145

[40] Buchon, N., Broderick, N. A., Chakrabarti, S. & Lemaitre, B. Invasive and indigenous microbiota impact intestinal stem cell activity through multiple pathways in *Drosophila*. *Genes Dev.* **23**, 2333–2344 (2009). 10.1101/gad.1827009 PMC2758745 19797770

[41] Bonfini, A., Liu, X. & Buchon, N. From pathogens to microbiota: How *Drosophila* intestinal stem cells react to gut microbes. *Dev. Comp. Immunol.* **64**, 22–38 (2016). 10.1016/j.dci.2016.02.008 26855015

[42] Brummel, T., Ching, A., Seroude, L., Simon, A. F. & Benzer, S. *Drosophila* lifespan enhancement by exogenous bacteria. *Proc. Natl. Acad. Sci. U. S. A.* **101**, 12974–12979 (2004). 10.1073/pnas.0405207101 PMC516503 15322271

[43] Clark, R. I. et al. Distinct shifts in microbiota composition during *Drosophila* aging impair intestinal function and drive mortality. *Cell Rep.* **12**, 1656–1667 (2015). 10.1016/j.celrep.2015.08.004 PMC4565751 26321641

[44] Li, H., Qi, Y. & Jasper, H. Preventing age-related decline of gut compartmentalization limits microbiota dysbiosis and extends lifespan. *Cell Host Microbe* **19**, 240–253 (2016). 10.1016/j.chom.2016.01.008 PMC5106289 26867182

[45] Sharon, G. et al. Commensal bacteria play a role in mating preference of *Drosophila melanogaster*. *Proc. Natl. Acad. Sci. U. S. A.* **107**, 20051–20056 (2010). 10.1073/pnas.1009906107 PMC2993361 21041648

[46] Schretter, C. E. et al. A gut microbial factor modulates locomotor behaviour in *Drosophila*. *Nature* **563**, 402–406 (2018). 10.1038/s41586-018-0634-9 PMC6237646 30356215

[47] Engel, P., Kwong, W. K. & Moran, N. A. *Frischella perrara* gen. nov., sp. nov., a gammaproteobacterium isolated from the gut of the honeybee, *Apis mellifera*. *Int. J. Syst. Evol. Microbiol.* **63**, 3646–3651 (2013). 10.1099/ijs.0.049569-0 23606484

[48] Tyler, A. D. *et al.* Evaluation of Oxford Nanopore’s MinION Sequencing Device for Microbial Whole Genome Sequencing Applications. *Sci. Rep.* **8**, (2018). 10.1038/s41598-018-29334-5 PMC6053456 30026559

[49] Zhang, T. *et al.* The newest Oxford Nanopore R10.4.1 full-length 16S rRNA sequencing enables the accurate resolution of species-level microbial community profiling. *Appl. Environ. Microbiol.* **89**, (2023). 10.1128/aem.00605-23 PMC10617388 37800969

[50] Abachin, E. et al. Formation of D-alanyl-lipoteichoic acid is required for adhesion and virulence of *Listeria monocytogenes*. *Mol. Microbiol.* **43**, 1–14 (2002). 10.1046/j.1365-2958.2002.02723.x 11849532

[51] Islam, M. S., Aryasomayajula, A. & Selvaganapathy, P. R. A review on macroscale and microscale cell lysis methods. *Micromachines* vol. 8 (2017).

[52] Rodríguez-Pérez, H., Ciuffreda, L. & Flores, C. NanoCLUST: A species-level analysis of 16S rRNA nanopore sequencing data. *Bioinformatics* **37**, 1600–1601 (2021). 10.1093/bioinformatics/btaa900 33079990

[53] Rodríguez-Pérez, H., Ciuffreda, L. & Flores, C. NanoRTax, a real-time pipeline for taxonomic and diversity analysis of nanopore 16S rRNA amplicon sequencing data. *Comput. Struct. Biotechnol. J.* **20**, 5350–5354 (2022). 10.1016/j.csbj.2022.09.024 PMC9522874 36212537

[54] Curry, K. D. et al. Emu: Species-level microbial community profiling of full-length 16S rRNA Oxford Nanopore sequencing data. *Nat. Methods* **19**, 845–853 (2022). 10.1038/s41592-022-01520-4 PMC9939874 35773532

[55] Bombin, A. et al. Influence of lab adapted natural diet and microbiota on life history and metabolic phenotype of *Drosophila melanogaster*. *Microorganisms* **8**, 1–27 (2020). 10.3390/microorganisms8121972 PMC7763083 33322411

[56] Marotz, C. et al. DNA extraction for streamlined metagenomics of diverse environmental samples. *Biotechniques* **62**, 290–293 (2017). 10.2144/000114559 28625159

[57] Thompson, L. R. et al. A communal catalogue reveals Earth’s multiscale microbial diversity. *Nature* **551**, 457–463 (2017). 10.1038/nature24621 PMC6192678 29088705

[58] Hug, L. A. et al. A roadmap for equitable reuse of public microbiome data. *Nat. Microbiol.* **10**, 2384–2395 (2025). 10.1038/s41564-025-02116-2 41006832

[59] Kumar, S. *et al.* MEGA12: Molecular Evolutionary Genetic Analysis Version 12 for Adaptive and Green Computing. *Mol. Biol. Evol.* **41**, msae263 (2024). 10.1093/molbev/msae263 PMC11683415 39708372

[60] Saitou, N. & Nei, M. The neighbor-joining method: a new method for reconstructing phylogenetic trees. *Mol. Biol. Evol.* 10.1093/oxfordjournals.molbev.a040454 (1987) 10.1093/oxfordjournals.molbev.a040454. 3447015

[61] Felsenstein, J. Confidence limits on phylogenies: An approach using the bootstrap. *Evolution* **39**, 783–791 (1985). 10.1111/j.1558-5646.1985.tb00420.x 28561359

[62] Nei, M. & Kumar, S. *Molecular Evolution and Phylogenetics* (Oxford University Press, Incorporated, 2000).

